# Patient Preferences for Technology-Assisted Patient-Reported Outcomes Measurement of Mental Health Symptoms Among Veterans: Cross-Sectional Survey

**DOI:** 10.2196/83149

**Published:** 2026-02-13

**Authors:** Bella Etingen, Eric J Richardson, Stephanie L Shimada, Jennifer A Palmer, Ndindam Ndiwane, Robin T Higashi, Felicia R Bixler, Bridget M Smith, Terry J Newton, Timothy P Hogan

**Affiliations:** 1 eHealth Partnered Evaluation Initiative VA Bedford Healthcare System Bedford, MA United States; 2 Research and Development Service Dallas VA Medical Center Dallas, TX United States; 3 Peter O'Donnell Jr. School of Public Health UT Southwestern Medical Center Dallas, TX United States; 4 Center for Health Optimization and Implementation Research (CHOIR) VA Boston Healthcare System Boston, MA United States; 5 Center for Health Optimization and Implementation Research (CHOIR) VA Bedford Healthcare System Bedford, MA United States; 6 Department of Health Law, Policy, and Management Boston University School of Public Health Boston, MA United States; 7 Division of Health Informatics and Implementation Science Department of Population and Quantitative Health Sciences UMass Chan Medical School Worcester, MA United States; 8 Section of General Internal Medicine Boston University's Chobanian & Avedisian School of Medicine Boston, MA United States; 9 Center of Innovation for Complex Chronic Healthcare (CINCCH) Edward Hines Jr. VA Hospital Hines, IL United States; 10 Northwestern University Feinberg School of Medicine Chicago, IL United States; 11 Office of Connected Care Veterans Health Administration US Department of Veterans Affairs Washington, DC United States

**Keywords:** measurement-based care, patient preferences, patient-reported outcomes, technology-based assessment, veterans

## Abstract

**Background:**

The Veterans Health Administration is promoting patient-reported outcome measure (PROM) collection for measurement-based mental health care. Understanding veteran preferences about how and when to complete PROMs is critical to support their implementation.

**Objective:**

We examined veteran preferences for timing and use of different technology platforms to complete mental health–related PROMs.

**Methods:**

We invited a national sample of 1373 veterans to complete a survey; 858 (62.5%) responded. Surveys asked about veteran preferences for how and when to complete mental health–related PROMs. We characterized responses using descriptive statistics and estimated multiple logistic regression models to examine associations between veteran demographic and health characteristics and preferences for completing PROMs.

**Results:**

Most veterans preferred completing PROMs between appointments (607/801, 75.8%) using features of a patient portal (410/801, 51.2%), during appointments (589/801, 73.5%) verbally (413/801, 51.6%), and while at the medical center (480/801, 59.9%) on paper (189/801, 23.6%) or a tablet computer (180/801, 22.5%). Hispanic (vs non-Hispanic) veterans had 3.32 (95% CI 1.04-10.58) times higher odds of preferring to complete PROMs at the medical center, and veterans with lower (vs higher) socioeconomic status had lower odds (odds ratio 0.61, 95% CI 0.40-0.93) of preferring to complete PROMs in between appointments but 1.97 (95% CI 1.23-3.16) times higher odds of preferring to complete PROMs during appointments.

**Conclusions:**

As the Veterans Health Administration and other health care systems seek to expand the integration of PROM data into health care services, adaptive and flexible approaches to PROM administration that align with patient preferences, including those that leverage technology platforms in the remote collection of these data, may bolster implementation. Our results indicate that such implementation efforts should consider patient ethnicity and socioeconomic status. Our findings further suggest that these efforts could benefit from incorporating PROM administration into online patient portals, developing mobile health apps that support PROM completion through patients’ personal devices in between clinical encounters, and engaging care team members in PROM administration during appointments.

## Introduction

Despite encouraging evidence of their clinical utility, the integration of patient-reported outcome measure (PROM) data into health care practice—also referred to as measurement-based care (MBC)—remains limited [1-5]. A recent Cochrane review of 116 randomized studies concluded that using PROM data in health care has a moderate effect on improving important aspects of clinical care, including accuracy of diagnoses, disease management, and patient-healthcare team member communication [4]. Nevertheless, only 60% of a recent sample of 600 hospital and clinic administrators reported wanting to implement routine PROM collection within their organization to improve patient experiences, and only 38% of the total sample indicated that they had done so [6].

There is recognition that PROM collection in the context of clinical care may be bolstered by leveraging technology platforms [7] either in between clinical encounters or before an encounter, while patients are waiting to see their care team members. In fact, several professional organizations have suggested that one strategy for bolstering implementation of MBC practices is to improve engagement by aligning PROM administration with patient needs and preferences [8,9]. However, additional information is needed regarding patient preferences for timing and use of different technology platforms to complete PROMs, particularly those related to potentially sensitive topics such as mental health–related symptoms.

To date, research regarding such patient preferences is mixed, largely relying on qualitative data or focused narrowly on specific contexts of care [10-12]. For example, one recent systematic review of 14 qualitative studies reported mixed findings among patients regarding preferred modality (paper-and-pencil vs electronic), timing (at home vs at the clinic), and frequency of completing PROMs as part of their health care [13]. This review further noted a range of reasons for patient preferences, including that patients who preferred to complete PROMs at home felt that they would be more likely to respond honestly, whereas patients who preferred to complete PROMs at the clinic liked that it gave them something to do while they waited for their appointment [13]. Other literature suggests that preferences may be influenced by demographic characteristics [14]; however, little research has been conducted regarding preferences for completing PROMs among diverse populations. Taken together, the existing literature base indicates that additional work focused on patient preferences for using technology platforms to complete PROMs is warranted.

The Veterans Health Administration (VHA), the largest integrated health care system in the United States, is currently working to further the integration of patient-generated health data (PGHD)—including PROMs—into practice; this is evidenced in part by national calls to incorporate MBC practices across mental health services and by the development of policies to guide veteran PGHD collection and use [15-17]. Tailoring the timing and mode of PROM administration, including through the use of relevant technology platforms, may increase the likelihood that veterans will share such data with their care team members.

Several technology platforms, including but not limited to VHA’s online patient portal (ie, My HealtheVet), the VHA automated text messaging system (ie, Annie), and platforms targeted toward the implementation of MBC in mental health services (ie, Behavioral Health Lab, Mental Health Assistant, and Mental Health Checkup [18-21]), have been available within the VHA health care system to support the administration of PROMs and their integration into care. Taken together, these various technology platforms allow for the collection of PROMs in numerous ways, including having patients complete PROMs before a clinical encounter from outside of the clinic, before a clinical encounter while in a clinic waiting room, or verbally with a member of their care team. Depending on how PROM data are collected, patients may enter responses directly into one of these platforms, or their care team members may either enter the data or add text to a clinical note in the electronic health record. The objective of this analysis was to characterize veteran preferences for timing and use of different technology platforms to complete mental health–related PROMs and assess factors associated with preferences regarding when (eg, in between, during, or just before clinical encounters) to complete mental health–related PROMs.

## Methods

### Design

These data were collected as part of a longitudinal survey effort initiated in 2017 and referred to as the Veterans Engagement With Technology Collaborative (VET-C) cohort. The overall goal of the VET-C cohort was to collect veteran feedback on VHA patient-facing technologies intended to improve access to and delivery of care and support veteran self-management [22]. The VET-C cohort was initiated by the VHA’s Office of Connected Care in partnership with investigators from the VHA’s Quality Enhancement Research Initiative program. Survey data were combined with additional demographic and health information from VHA administrative data. Of note, surveys were reviewed by a veteran before their fielding for readability and relevance.

### Sample

A set of 14 geographically dispersed VHA facilities were identified as VET-C recruitment sites. We identified veterans who were active users of VHA patient-facing technologies (ie, secure messaging) to be included in the cohort. Secure messaging use was defined as sending a minimum of 5 messages through the VHA’s online patient portal in the year before cohort recruitment. Further details related to sampling for the VET-C cohort can be found in previous publications [22,23].

### Procedures

Survey data were collected from members of the VET-C cohort at 3 time points: 2017 to 2018, 2019 to 2020, and 2021 to 2022. Data collection procedures for the first 2 rounds of surveys are described in previous publications [22,23]. Veterans who responded to the first 2 surveys were invited to complete the third survey, which was administered via mail. Nonresponders to the initial mailing were mailed a follow-up survey approximately 4 weeks later to enhance response rates. Veterans were also provided the option to complete the survey via phone with a member of our evaluation team; 2 veterans chose to do so. Survey data were entered into the VHA’s secure electronic data capture platform, REDCap (Research Electronic Data Capture) [24].

### Measures

The survey collected data on veteran demographic characteristics, health and health care use, technology ownership and use, and preferences for completing PROMs. Demographic characteristics included veteran age, sex, race, ethnicity, source of health care (ie, within or outside the VHA), level of difficulty paying for basics such as food and heating and cooling, and current housing situation. We asked veterans to rate their physical and mental health on a 5-point Likert scale ranging from excellent to poor. Veteran health conditions were obtained from diagnosis codes in the VHA Corporate Data Warehouse. We also used Corporate Data Warehouse data to fill in any missing survey data on key demographic variables.

To assess veteran preferences for completing mental health–related PROMs, we provided basic framing language to explain the purpose of PROMs: “Now we would like to know your preferences for providing information about your health to your VHA healthcare team. Sometimes, your VHA healthcare team members ask you to complete questionnaires about your symptoms or health status (these are called assessments). We are interested in how and when you prefer to complete such assessments.” Following this framing, we asked veterans to report both when and how they preferred to complete PROMs (ie, “When and how do you prefer to complete assessments about your behavioral health (eg, depression, anxiety, alcohol use, stress)?”). Regarding preferences related to *when*, response options were “in between my appointment when I am not at the VHA,” “when I am at the VHA,” and “during my appointments with my VHA provider(s).” Regarding preferences related to *how*, response options included the use of different technology platforms: (1) secure messaging, a mobile health app, or mailed paper-and-pencil assessments for in between appointments; (2) on a kiosk, on paper-and-pencil, or on a tablet computer when at the VHA (in the waiting room before an appointment); and (3) on a tablet computer, on a kiosk, on paper-and-pencil, or verbally with their providers during appointments. Veterans were allowed to select more than one response per question, and we included all responses in our data analyses.

### Analyses

We characterized veteran survey responses using descriptive statistics. We then used multiple logistic regression models to examine factors associated with veteran preferences regarding when to complete mental health–related PROMs, controlling for key demographic characteristics and health conditions. All statistical analyses were performed using Stata/MP (version 17; StataCorp).

### Ethical Considerations

This work was reviewed by the institutional review boards at the VHA hospital in Hines, Illinois, and the VHA Bedford Healthcare System in Bedford, Massachusetts, and designated as program evaluation for quality improvement purposes, exempting it from further oversight (VHA Handbook 1058.05). Returning a survey was considered consent to participate in the evaluation. All data were deidentified for analysis. No compensation was provided to participants.

## Results

### Response Rate

We mailed surveys to 1373 veterans who completed rounds 1 and 2 surveys from the VET-C cohort and asked them to complete a third-round survey. Of the 1373 third-round survey invitations, 10 (0.7%) were returned due to bad addresses, and an additional 5 (0.4%) were returned because the veteran was deceased. From the sample of 1358 veterans who were thus eligible to complete a round 3 survey, we received responses from 858 (63.2%). We included veterans with complete data on model covariates in our analyses (801/858, 93.4%).

### Sample Characteristics

Most survey respondents were aged >65 years (603/858, 70.3%), male (751/858, 87.5%), White (763/858, 88.9%), and married or in a civil union (582/827, 70.4%) and received their health care mostly from the VHA (617/857, 72.0%; Table 1). Nearly half (413/852, 48.5%) reported having obtained a master’s degree or higher professional degree. Most reported being in excellent, very good, or good physical (596/854, 69.8%) and mental (695/856, 81.2%) health. The vast majority (807/837, 96.4%) lived in their own house or apartment. Approximately a quarter (189/776, 24.4%) of the sample reported low socioeconomic status (SES; ie, difficulties paying for basics such as food and heating and cooling). Nearly half (404/858, 47.1%) had a diagnosis consistent with an anxiety disorder, 38.1% (327/858) had a diagnosis consistent with a depressive disorder, and 29.3% (251/858) had a posttraumatic stress disorder diagnosis.

**Table 1:** Veteran characteristics.

Characteristic			Participants, n/N (%)
**Age (years)**			
	>65	603/858 (70.3)	
	≤65	255/858 (29.7)	
**Sex**			
	Male	751/858 (87.5)	
	Female	107/858 (12.5)	
**Race**			
	Black, African American, or other	95/858 (11.1)	
	White	763/858 (88.9)	
**Hispanic or Latino Ethnicity**			
	No	831/858 (96.9)	
	Yes	27/858 (3.1)	
**Relationship status**			
	Married or in a civil union	582/827 (70.4)	
	Neither married nor in a civil union	245/827 (29.6)	
**Educational level**			
	High school graduate or lower	106/852 (12.4)	
	At least some college or vocational education (1-4 y)	333/852 (39.1)	
	Master’s, professional, or doctoral degree	413/852 (48.5)	
**Place where they received medical care**			
	Mostly at the VHA^a^	617/857 (72.0)	
	Other	240/857 (28.0)	
**Physical health**			
	Excellent, very good, or good	596/854 (69.8)	
	Fair or poor	258/854 (30.2)	
**Mental health**			
	Excellent, very good, or good	695/856 (81.2)	
	Fair or poor	161/856 (18.8)	
**Housing**			
	Own or rent an apartment or house	807/837 (96.4)	
	Other	30/837 (3.6)	
**Financial difficulties**			
	No	587/776 (75.6)	
	Yes	189/776 (24.4)	
**Mental health condition**			
	Anxiety disorders	404/858 (47.1)	
	Depression	327/858 (38.1)	
	Posttraumatic stress disorder	251/858 (29.3)	

^a^VHA: Veterans Health Administration.

### Veteran Preferences for PROM Completion

Most veterans in our sample preferred completing mental health–related PROMs in between appointments when not at the VHA (607/801, 75.8%) or during appointments with their VHA providers (589/801, 73.5%; Table 2). Respondents endorsed to a lesser degree the option to complete PROMs when at the VHA before an appointment (480/801, 59.9%). Regarding preferences for how to complete PROMs, most veterans reported similar preferences for completing mental health–related PROMs: (1) using a secure messaging feature through a patient portal when completing these PROMs in between appointments when not at the VHA (410/801, 51.2%), (2) on paper-and-pencil (189/801, 23.6%) or on a tablet computer (180/801, 22.5%) when completing PROMs at the VHA before an appointment, and (3) verbally (413/801, 51.6%) when completing PROMs during appointments with their VHA providers.

**Table 2:** Veteran preferences for completing patient-reported outcome measures about mental health symptoms (N=801).

Preference		Participants, n (%)
**In between my appointments when I am not at the VHA^a^**		607 (75.8)
	Using secure messaging through a patient portal	410 (51.2)
	Using a mobile health app on a smartphone, tablet, or other computer	155 (19.4)
	Completing paper-and-pencil assessments mailed to me	132 (16.5)
**During my appointments with my VHA providers**		589 (73.5)
	Verbally	413 (51.6)
	On a tablet computer	119 (14.9)
	On paper-and-pencil	104 (13.0)
	On a kiosk	48 (6.0)
**When I am at the VHA**		480 (59.9)
	On paper-and-pencil in the waiting room before my appointment	189 (23.6)
	On a tablet computer in the waiting room before my appointment	180 (22.5)
	On a kiosk in the waiting room before my appointment	152 (19.0)

^a^VHA: Veterans Health Administration.

### Factors Associated With Veteran Preferences for When to Complete PROMs

When controlling for demographic and health-related variables, veterans of Hispanic (vs non-Hispanic) ethnicity had greater odds of preferring to complete mental health–related PROMs (odds ratio [OR] 3.32, 95% CI 1.04-10.58) when at the VHA before an appointment (Table 3). Veterans reporting having (vs not having) financial difficulties had lower odds of preferring to complete mental health–related PROMs in between appointments when not at the VHA (OR 0.61, 95% CI 0.40-0.93) but greater odds of preferring to complete such PROMs during a visit with their VHA providers (OR 1.97, 95% CI 1.23-3.16).

**Table 3:** Factors associated with preferences for when to complete patient-reported outcome measures about mental health symptoms.

Factor			In between appointments when not at the VHA^a^, OR^b^ (95% CI)		At the VHA, OR (95% CI)		During appointments, OR (95% CI)
**Age (years)**							
	>65 (reference: ≤65)	1.35 (0.88-2.06)		1.09 (0.75-1.58)		1.21 (0.80-1.80)	
**Sex**							
	Female (reference: male)	1.35 (0.74-2.48)		1.28 (0.76-2.15)		1.05 (0.60-1.85)	
**Race**							
	Black or other (reference: White)	0.69 (0.37-1.29)		0.87 (0.50-1.51)		0.69 (0.38-1.25)	
**Hispanic or Latino Ethnicity**							
	Hispanic (reference: non-Hispanic)	1.36 (0.45-4.13)		*3.32*^c^ (1.04-10.58)		3.27 (0.88-12.21)	
**Marital status**							
	Married (reference: not married or in a civil union)	0.77 (0.50-1.20)		0.93 (0.64-1.34)		0.91 (0.60-1.39)	
**Educational level**							
	At least some college or vocational education (reference: high school or lower)	0.83 (0.47-1.49)		0.87 (0.53-1.44)		0.61 (0.33-1.13)	
**Place where care is received**							
	Mostly at the VHA (reference: other)	0.96 (0.65-1.43)		0.86 (0.61-1.21)		0.97 (0.65-1.42)	
**Physical health**							
	Excellent, very good, or good (reference: fair or poor)	0.79 (0.51-1.21)		0.96 (0.67-1.38)		0.96 (0.63-1.45)	
**Mental health**							
	Excellent, very good, or good (reference: fair or poor)	0.62 (0.36-1.07)		1.10 (0.70-1.72)		1.46 (0.88-2.41)	
**Housing status**							
	Owning or renting (reference: other)	0.95 (0.32-2.80)		1.22 (0.50-2.96)		1.74 (0.67-4.53)	
**Financial difficulties**							
	Yes (reference: no)	*0.61*^c^ (0.40-0.93)		1.34 (0.91-1.97)		*1.97*^d^ (1.23-3.16)	
**Depression**							
	Yes (reference: no)	1.46 (0.92-2.30)		1.23 (0.83-1.83)		1.14 (0.73-1.78)	
**Anxiety**							
	Yes (reference: no)	1.37 (0.84-2.24)		0.81 (0.53-1.23)		0.97 (0.61-1.55)	
**PTSD^e^**							
	Yes (reference: no)	0.67 (0.41-1.11)		1.25 (0.82-1.91)		1.32 (0.81-2.14)	

^a^VHA: Veterans Health Administration.

^b^OR: odds ratio.

^c^*P*<.05.

^d^*P*<.01.

^e^PTSD: posttraumatic stress disorder.

## Discussion

### Principal Findings

Overall, veterans indicated a preference for completing mental health–related PROMs either in between or during appointments with their VHA providers as opposed to before appointments in clinic waiting rooms and preferred to do so using secure messaging via a patient portal or verbally with their providers, respectively. However, our results suggested differences in preferences based on demographic characteristics, including ethnicity and SES. These findings suggest that technology platforms, including secure messaging features of online patient portals, may bolster implementation of remote MBC practices, although the rollout of such remote MBC practices may benefit from targeted improvement efforts among certain segments of the patient population, including those patients experiencing financial stressors. Our findings further suggest that care team members should elicit preferences for how and when to complete PROMs from each veteran and administer PROMs in accordance with those preferences to optimize the likelihood that veterans will provide PROM data as part of their health care. These results present a foundation through which the VHA, as well as other health care systems, can support patient-centered implementation of MBC practices and use of PGHD in clinical care.

Patient-facing technologies such as patient portals may facilitate completion of PROMs for MBC; however, more research is needed to understand when patients are willing to use technologies to complete PROMs and when they are not. Our findings suggest a high degree of comfort with completing PROMs through secure messaging via a patient portal among our cohort, which may be due in part to the fact that all our survey respondents had used secure messaging at some point. However, there is high engagement overall among veterans with the VHA’s online patient portal, My HealtheVet [25,26]. As secure messaging was first implemented in the VHA nationwide in 2010, its use has continued to grow [25]. In May 2025 alone, the number of secure messages sent by veterans exceeded 1.7 million [27]. The literature also suggests that secure messaging has been used in the VHA to support MBC for behavioral health [26]. As such, facilitating PROM completion via secure messaging may bolster engagement in MBC practices among some veterans.

In addition, the literature has reported that older veterans are less likely than younger veterans to prefer using smartphone or tablet technologies [28,29]. Thus, the average age of our overall sample may be one reason for the low rate of reported preferences for using smartphone or tablet technologies to complete PROMs [30]. Alternatively, the low preferences for use of smartphone or tablet technologies, even in a sample of individuals who are known technology users, may suggest that veterans are more trusting of the security and privacy associated with other methods for completing PROMs, such as secure messaging through a patient portal. However, several studies in the context of cancer care have found that integration of smartphone apps with tethered patient portals increases patient engagement in completing PROMs [31]. As the use of health-related mobile apps increases, the ability to synchronize mobile app data with patient portals may facilitate veteran use of apps to collect and share data with the VHA [32,33].

Our findings further highlight the need to be sensitive toward differences across the demographic characteristics (eg, ethnicity and SES) of patients to ensure opportunity to complete PROMs. In line with previous research, we observed differences in preferences among certain segments of the veteran population that could contribute to the ongoing digital divide in health care access and outcomes [14,34-36]. While we are still unsure of the extent, there seems to be a strong preference among Hispanic veterans to complete assessments while present at the VHA. This is aligned with prior research suggesting that portal users of Hispanic ethnicity were less likely than users of White race to use secure messaging [37]. Similarly, in a 2007 nationwide survey on health IT use, respondents of Hispanic ethnicity were less likely to access technologies between appointments with providers [38]. This could be due in part to reduced access to technology at home, lower health or computer literacy, or other health management and health communication preferences [39,40]. Patients’ prior experiences with PROM completion or use of technology for health may also at least in part drive their preferences regarding when and how to complete PROMs. Although our data do not speak to these points, future work exploring such relationships is warranted.

In addition, veterans in our sample who reported experiencing financial difficulties were less likely to prefer completing PROMs in between appointments. This may be due in part to lack of access to technology with which to complete PROMs remotely, lower technological literacy, or lower health literacy [41,42]. Studies have shown that patients with lower SES are more likely to have privacy concerns or report not trusting remote measurement options [34]. This is supported in part by the preferences reported by the veterans in our sample for completing assessments verbally with their providers.

Taken together, our findings can serve as a foundation for health care systems to design and implement patient-centered procedures to collect PROMs, including using technology platforms to assist with remote PROM collection. As our results suggest, methods of administering PROMs cannot assume a one-size-fits-all approach. The most effective ways to collect PROMs may depend on patient technology preferences and the availability of different technology platforms in different health care system settings [43]. It may also depend on the cadence with which PROMs are collected in different clinical contexts. For example, PROMs collected as part of mental health care services, such as evidence-based psychotherapies widely offered within the VHA, where patients are seen frequently (often weekly), may be collected much more often than in other medical settings such as primary care, where patients are typically seen 1 or 2 times per year. The frequency with which PROMs are collected may influence patient preferences for how to complete those PROMs—one can imagine that patients who are asked to complete the same PROMs every week in tandem with evidence-based psychotherapy visits may prefer to complete those PROMs virtually, whereas patients who are asked to complete PROMs annually in tandem with primary care visits may prefer to complete those PROMs in the clinic waiting room or verbally with a member of their care team. Additionally, older veterans may not use smartphones or tablets but may be more amenable to secure messaging as an alternative for PROM completion.

Moreover, although we cannot assume that changing the way in which a validated PROM is administered will retain its psychometric properties (eg, if a measure is validated to be administered on paper-and-pencil, having a health care team member verbally ask the questions may impact the psychometric properties of the measure), several reviews of the literature have concluded that different methods (eg, electronic vs paper-and-pencil) and even modest adaptations to collection procedures (eg, interactive voice response systems) do not reduce the validity of the results [44-46]. Thus, the ability to meet veteran preferences does not seem to reduce data quality and, in fact, may potentially improve PROM data by facilitating more genuine responses. Future research may examine more fully the extent to which the psychometric properties of PROMs are impacted based on the mode of administration used.

### Limitations

Several limitations need to be considered when interpreting our results. First, our sample comprised veterans who were known users of patient-facing technologies, which may impact generalizability. Second, the homogeneity of our sample demographics (ie, mostly White, male, and aged >65 years) may limit the generalizability of our findings. Third, our relatively small sample size may have limited the statistical power of our analyses. Fourth, when completing surveys, there is the risk of recall and response biases. In addition, our data do not address patient perceptions of the usability of the various technology platforms that may be used to administer PROMs, a factor that could influence preferences and that should be assessed in future work. Finally, due to the nature of the cross-sectional design of the data collection, we cannot make any inferences about the causality between veteran characteristics and preferences for completing PROMs. Despite these limitations, these findings add important insights to our understanding of patient preferences for completing PROMs in different contexts and using different technology platforms.

### Conclusions

As the VHA seeks to expand initiatives related to the use of MBC practices and PGHD as part of health care services, adaptive and flexible approaches to PROM administration that align with veteran preferences [47] and leverage technology platforms to collect PROMs remotely [7] may be beneficial. Our findings provide foundational insights into veteran preferences for completing PROMs and can be used to design patient-centered PROM data collection procedures. Furthermore, our findings suggest that initiatives focused on promoting the administration of PROMs as part of clinical care could incorporate administering PROMs using the VHA’s online patient portal, developing mobile health apps that support veteran completion of PROMs using their personal devices in between VHA clinical encounters and building veteran trust in the privacy and security of those apps, and engaging care team members in the administration of PROMs during appointments.

## Abbreviations

MBC: measurement-based care
OR: odds ratio
PGHD: patient-generated health data
PROM: patient-reported outcome measure
REDCap: Research Electronic Data Capture
SES: socioeconomic status
VET-C: Veterans Engagement With Technology Collaborative
VHA: Veterans Health Administration

## Data Availability

The datasets generated or analyzed during this evaluation are not publicly available because they are not permitted to leave the Veterans Health Administration (VHA) firewall without a data use agreement but are available from the corresponding author on reasonable request and with appropriate documentation.
